# Unveiling the Potential of Fish Swim Bladder–Derived Biomaterials in Urological Care

**DOI:** 10.1155/aiu/7996782

**Published:** 2026-10-12

**Authors:** Johannes Cansius Prihadi, Havian Daulung Telium, Pradipta Auwrel Putra, Alvin Chandra

**Affiliations:** ^1^ Division of Clinical Medicine Faculty of Medicine and Nutrition IPB University Bogor Indonesia ipb.ac.id; ^2^ Department of Surgery Division of Urology Atma Jaya Hospital Jakarta Indonesia; ^3^ School of Medicine and Health Sciences Atma Jaya Catholic University of Indonesia Jakarta Indonesia atmajaya.ac.id

**Keywords:** biomaterials, fish swim bladder, marine-derived collagen, urological reconstruction, urological tissue engineering

## Abstract

Fish swim bladder (FSB)–derived biomaterials have recently emerged as a promising marine‐based extracellular matrix (ECM) platform with potential applications in urological tissue engineering and regenerative medicine. This narrative review synthesizes current evidence regarding the structural, biological, and functional properties of FSB and evaluates its potential role in urological reconstruction. This narrative review was conducted using the PubMed, Google Scholar, and ScienceDirect databases with predefined keywords related to FSB‐derived biomaterials. Articles published from January 1, 2000, to December 31, 2025, were included. Eligible studies comprised original research, in vitro and in vivo experimental studies, and review articles. FSB is characterized by a high content of type I collagen, elastin, and glycosaminoglycans (GAGs), forming a well‐organized, hydrated, and mechanically resilient matrix that closely resembles native mammalian ECM. These properties support cellular adhesion, proliferation, and tissue regeneration, while also modulating inflammatory responses during wound healing. Preclinical studies demonstrate that decellularized FSB scaffolds exhibit favorable mechanical properties, biocompatibility, and the ability to promote constructive tissue remodeling. In addition, hydrolyzed derivatives, such as gelatin and collagen peptides, have demonstrated antioxidant and regenerative properties. However, intrinsic antimicrobial activity remains limited, often requiring additional functionalization to achieve clinically relevant antibacterial effects. Safety considerations, including potential allergenicity, variability in raw materials, and the risk of microbial contamination, underscore the importance of standardized processing and rigorous sterilization protocols. Composition, thermal stability, and gelation behavior vary substantially across fish species, indicating that species selection is a critical determinant of scaffold performance. Despite encouraging findings in experimental models, clinical translation in urological applications remains limited. Further research focusing on organ‐specific applications, long‐term safety, and optimized scaffold design is essential. Overall, FSB‐derived biomaterials represent a sustainable and versatile platform with significant potential to advance regenerative strategies in urological care.

## 1. Introduction

The urinary tract plays an essential role in maintaining human health; however, its structural and functional integrity is often disrupted by congenital anomalies, diseases, or traumatic injuries [1]. These conditions impose a significant clinical and socioeconomic burden, contributing to patient morbidity, reduced quality of life, and increased healthcare costs [1–4]. Pathologies requiring urological reconstruction range from life‐threatening bladder rupture to chronic disorders, such as urethral stricture, leading to renal impairment [5]. Despite ongoing advancements in surgical techniques, the search for an ideal reconstructive material remains a significant challenge in urology [6]. Currently, the gold standard for urological reconstruction relies on autologous grafts, such as buccal mucosa, which offer excellent biocompatibility and optimal vascular integration [7–9]. However, donor site morbidity—including pain, infection, and oral dysfunction—along with limited tissue availability, restricts their use in extensive reconstructions [10, 11]. In addition, the exposure of nonurological tissues (e.g., gastrointestinal tissue) and urine can lead to complications such as metabolic disturbances, stone formation, and chronic infection [12].

Tissue engineering has emerged as a promising alternative to overcome these limitations [13]. This multidisciplinary field integrates scaffolds, cells, and bioactive molecules to create living, functional tissues capable of supporting proper urological regeneration [14]. In urology, such innovations may lead to the development of biocompatible, “off‐the‐shelf” grafts for urethral, ureteral, and bladder reconstruction, potentially reducing dependence on donor tissue while improving functional outcomes [6]. Within the realm of tissue engineering, the choice of scaffold material is paramount [15]. While synthetic polymers and mammalian‐derived tissues have been extensively studied, a novel and highly promising frontier has opened in the form of marine‐derived biomaterials [15]. These materials, sourced from marine organisms, offer several distinct advantages over their terrestrial counterparts [16]. A primary benefit is the significantly reduced risk of zoonotic disease transmission [16].

One potential biomaterial source is collagen or gelatin derived from the fish viscera, specifically the swim bladder, which is known to have high bioactivity, hydrogel‐forming ability, and a promising safety profile [17, 18]. Collagen isolated from fish swim bladders (FSBs) (e.g., sturgeon) presents several attractive features such as high thermal stability, rapid gelation forming oriented fiber networks, favorable mechanical properties, and potentially reduced zoonotic infection risk compared to mammalian sources [19]. Furthermore, marine‐derived biomaterials can bypass the religious and cultural restrictions associated with the use of bovine or porcine tissues, making them a more globally acceptable option [20]. The fishing and aquaculture industries generate large amounts of swim bladders that are often discarded as waste [15]. The ability to convert this waste into high‐value medical products presents an opportunity to develop a sustainable, cost‐effective, and scalable source of raw materials [21].

Global aquatic animal production in 2022 reached 185 million tonnes, representing a 4% increase from 2020 [22]. Over the past decades, the worldwide consumption of aquatic animal food has risen substantially, progressing at a faster annual rate than the overall population growth [22]. In 2022, China accounted for the largest share of aquatic animal production (36%), followed by India (8%), Indonesia (7%), Vietnam (5%), and Peru (3%). Overall, these five nations contributed approximately 59% of global aquatic animal production [22]. By‐products from fish processing can account for up to 75% of the total fish weight [18]. Among these by‐products, fish organs and their components represent a particularly generative and underutilized resource with promising applications in biomedical and pharmaceutical fields [18, 23].

The FSB has recently gained attention as a nonmammalian biomaterial rich in type I collagen, elastin, and polysaccharides, and has been explored for multiple biomedical applications [17]. FSB‐derived biomaterials have shown promise in skin wound healing, dural repair, small‐vessel cardiovascular grafts, and in various applications—including food, cosmetics, and pharmaceuticals [17]. This narrative review aims to evaluate the potential of FSB‐derived biomaterials in three critical aspects: wound healing, antimicrobial effects, and safety. To date, there is limited published evidence of FSB‐based biomaterials being applied in the urology field. The findings of this review suggest that FSB‐based biomaterials could serve as a viable alternative and help guide future clinical applications and innovation in the urology field.

## 2. Materials and Methods

We performed a search (Table 1) using the PubMed, Google Scholar, and ScienceDirect databases with relevant keywords and their synonyms, including “fish swim bladder,” “fish bladder,” “fish,” “swim bladder,” “FSB,” “marine‐derived collagen,” “biomaterials,” “tissue engineering,” “collagen,” “urology,” “urinary tract,” “kidney,” “ureter,” “urinary bladder,” “urethra,” “injury,” “trauma,” “stricture,” “wound healing,” “angiogenesis,” “infections,” “antimicrobial,” “reconstruction,” “regeneration,” “mechanical,” “antioxidants,” “oxidative stress,” “allergy,” and “allergens.” The search was limited to articles published from January 1, 2000, to December 31, 2025. Eligible studies included original articles, in vitro and in vivo experimental studies, and review articles. We excluded articles that did not meet these eligibility criteria.

**TABLE 1:** The search strategy summary.

Items	Specification
Date of search	January 1, 2026

Databases and other sources searched	PubMed, Google Scholar, and ScienceDirect

Search terms used	“fish swim bladder,” “fish bladder,” “fish,” “swim bladder,” “FSB,” “marine‐derived collagen,” “biomaterials,” “tissue engineering,” “collagen,” “urology,” “urinary tract,” “kidney,” “ureter,” “urinary bladder,” “urethra,” “injury,” “trauma,” “stricture,” “wound healing,” “angiogenesis,” “infections,” “antimicrobial,” “reconstruction,” “regeneration,” “mechanical,” “antioxidants,” “oxidative stress,” “allergy,” and “allergens”

Timeframe	January 1, 2000–December 31, 2025

Inclusion and exclusion criteria	Inclusion criteria: • English‐language articles • Published original articles, in vitro and in vivo experimental studies, and review articles • Focus on the biological properties, mechanisms, safety, and potential clinical applications of fish swim bladder–derived biomaterials, particularly to support the context of urological tissue repair and regeneration Exclusion criteria: • Non‐English language articles • Case reports, case series, editorials, letters to the editor, and abstracts

Selection process	The first author conducted the selection, and all the coauthors approved the included studies

## 3. Fish Swim Bladder–Derived Biomaterials: Structure, Composition, and Mechanical Properties

### 3.1. Structure and Composition

The swim bladder is a gas‐filled organ located dorsally within the body cavity of most teleost fish, serving a critical role in buoyancy regulation and maintaining hydrostatic equilibrium [24]. It typically constitutes about 5% of the fish’s total body volume [17]. Anatomically, FSB consists of two chambers, anterior and posterior, separated by the ductus communicans (Figure 1) [17, 24]. The major structural components include type I collagen, which forms strong fibrillar networks in the tunica externa, and elastin, abundant in the muscularis mucosa [17]. Polysaccharides comprise approximately 10% of the swim bladder’s weight, primarily consisting of chondroitin sulfate (CS) and a small proportion of hyaluronic acid (HA) [17]. The tissue also incorporates inorganic minerals, predominantly calcium, together with potassium and magnesium, and trace elements such as zinc, copper, iron, and selenium, all of which are essential for physiological functions in humans [17].

**FIGURE 1:**
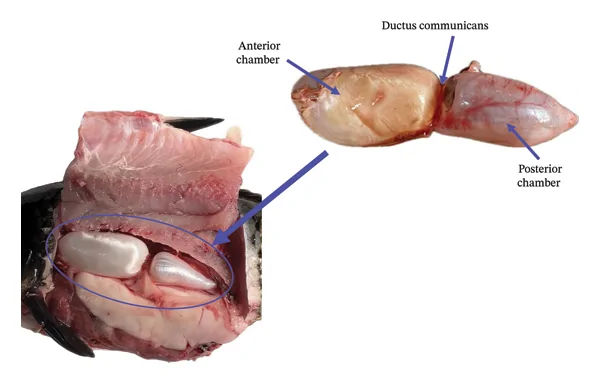
Fish swim bladder anatomy.

On a molecular level, the collagen structure closely resembles that of bovine pericardium, consisting of a triple‐helical arrangement of two *α*1 and one *α*2 chains, rich in glycine (approximately 30%), proline, hydroxyproline, threonine, methionine, isoleucine, and phenylalanine [17]. The collagen has been reported to exhibit a thermal denaturation point of approximately 35°C, which is slightly lower than that of mammalian collagen but higher than that of most fish organs, making it favorable for biomedical applications [17]. Its stability is maintained by hydrogen bonding and intermolecular crosslinks of glycerin triplets, while proline conformations preserve flexibility [17]. Extraction techniques—including pepsin dissolution, acid‐base dissolution, and fermentation—can yield between 30% and 60% collagen, depending on the fish species and processing method [17].

Additionally, the swim bladder is highly specialized, featuring a complex neuromuscular and vascular system [24]. Phalloidin staining has identified sphincter muscle cells at the pneumatic duct–esophagus junction and prominent muscular bands along the anterior chamber’s ventral surface, as well as bilaterally in the posterior chamber [24]. At the same time, thinner layers appear in other regions. A rete‐like vascular network runs mainly in the posterior chamber, ensuring countercurrent multiplication of blood gases [24]. Dense innervation has been demonstrated using markers such as zn‐12, SV2, and acetylcholinesterase [24]. Cholinergic fibers (i.e., choline acetyltransferase‐IR) are concentrated at the duct junction, whereas sympathetic fibers (i.e., tyrosine hydroxylase‐IR) are dispersed throughout the musculature and vasculature [24, 25].

The swim bladder is a specialized membranous organ primarily composed of type I collagen, which forms a densely organized fibrillar network [17]. This structure is complemented by the presence of elastic fibers and polysaccharides [17]. The collagen content of the swim bladder ranges from 35% to 90% of the dry weight, and moisture content ranges from 10% to 57%, with the combined collagen and moisture often exceeding 90%, making it both structurally robust and highly hydrated [26]. Amino acid analysis revealed a profile rich in glycine (33%–35%), proline (12%–18%), and hydroxyproline (8%–9%) [26]. Structural characterization using thermal and spectroscopic techniques, such as differential scanning calorimetry (DSC), attenuated total reflectance–Fourier transform infrared spectroscopy (ATR‐FTIR), and X‐ray diffraction (XRD), confirms the integrity of the triple helix, and atomic force microscopy (AFM) showed fibrillar periodicity between 70 and 77 nm [26]. Cruz‐López et al. compared the moisture content of raw swim bladder tissue in many fish species, ranging from approximately 67%–83%, which may result from alterations in swim bladder hydration associated with processing and storage, as well as underlying biological variability [27].

### 3.2. Mechanical Properties

The native FSB is a dense, fibrillar collagen membrane that is mechanically anisotropic, with both the tensile strength and elastic modulus higher in the circumferential direction than in the axial direction [17, 28]. This directional behavior reflects the underlying orientation of collagen fiber bundles [17]. An in vitro study by Han et al. demonstrated that native FSB tissue exhibited a tensile strength of 6.82 MPa and an elastic modulus of 31.18 MPa, reflecting the fully intact and hydrated collagen fiber network of unprocessed swim bladder [28]. Overall, native FSB retained the second‐highest tensile strength and a mid‐range elastic modulus among the decellularized scaffold groups [28]. Notably, the elastin‐rich composition of the FSB confers an elastic modulus that compares favorably with that of bovine pericardium, a tissue already validated for cardiovascular and reconstructive applications [17], indicating that FSB‐derived matrices can provide adequate compliance alongside structural support.

Chemical crosslinking is a key modifiable determinant of FSB mechanical performance. An experimental study by Zhang et al. showed that uncrosslinked FSB membranes exhibit a tensile strength of approximately 7.09 MPa, whereas 3‐(3‐dimethylaminopropyl)‐1‐ethylcarbodiimide hydrochloride (EDC)/N‐hydroxysuccinimide (NHS)‐crosslinked FSB reaches approximately 24.71 MPa, with a corresponding increase in denaturation temperature, indicating that crosslinking density can be tuned to meet the strength requirements of the intended surgical site without sacrificing biocompatibility [29]. Comparable findings have been reported by Yin et al. who demonstrated that crosslinked acellular swim bladder collagen membranes achieved an increase in circumferential fracture tensile stress to approximately 25.66 MPa from approximately 5.75 MPa and in elastic modulus to approximately 184.43 MPa from approximately 61.42 MPa, as well as an increase in axial fracture tensile stress to approximately 10.18 MPa from approximately 3.05 MPa and in elastic modulus to approximately 82.86 MPa from approximately 27.01 MPa [30]. Collectively, these findings indicate that, beyond their baseline structural robustness, FSB‐derived matrices possess a tunable mechanical profile that can be calibrated through processing strategy to suit a range of reconstructive demands.

These mechanical characteristics carry direct implications for urologic surgery, where grafts must withstand suturing, intraluminal pressure, and repeated distension without failure. An experimental study by Bai et al. using Sprague–Dawley rats proved that decellularized FSB patches and tubes exhibited burst pressure and suture retention comparable to native swim bladder tissue and superior to those of decellularized rat thoracic aorta [31]. For context, native human bladder tissue had been biomechanically characterized through uniaxial tensile testing in both sexes, yielding sex‐specific reference values. Martins et al. reported that female bladder tissue exhibited a mean maximum tensile stress of approximately 0.9 MPa (maximum stress ranged from 0.5 to 2.6 MPa) under uniaxial tensile testing [32]. In a male bladder, Berardo et al. reported that the uniaxial tensile stress yielded a median of 0.47 MPa (IQR 0.44–0.54 MPa) and an elastic modulus of 1.40 MPa (IQR 1.16–1.58 MPa) [33], while Johnson et al. reported that failure stress and elastic modulus demonstrated strain rate dependency, reaching a maximum failure stress of 0.65 MPa and an elastic modulus of 2.16 MPa at a strain rate of 500%/s [34]. In another study, Rassoli et al. reported that the tensile strength of human ureter was approximately 2.31 MPa in the circumferential direction and 3.48 MPa in the longitudinal direction [35].

Adequate suture retention is similarly critical for anastomotic integrity (e.g., in urethroplasty and ureteral anastomosis). Crosslinked FSB as surgical sutures had been shown by Luan et al., where in the dry state (i.e., in the oven at 30 °C for 5  h), decellularized crosslinked single‐strand swim bladder (DCSS) suture reached a peak stress of 450 MPa versus 340 MPa for catgut, despite DCSS suture having a lower maximum strain (28%) than catgut (39%), indicating DCSS suture was stiffer and stronger but less extensible [36]. In the wet state (soaked for 30 minutes in PBS solution), the tensile stress of DCSS suture did not reduce significantly, whereas catgut’s tensile stress dropped by approximately 50 MPa [36]. Fracture energy of DCSS suture was significantly higher than that of catgut in both the dry and wet states, meaning that DCSS suture can absorb more mechanical energy before failure [36]. Another comparison in the wet state showed that the decellularized crosslinked double‐strand swim bladder (DCDS) suture achieved a breaking force and fracture energy more than 2‐fold higher than those of the DCSS suture [36]. Maximum strain was also significantly higher in the DCDS suture (35%) than in the DCSS suture (24%) in the wet state, indicating that the double‐strand configuration conferred both greater toughness and extensibility [36].

The mechanical profiles of FSB‐derived biomaterials across their processed forms, such as membrane, patch, tube, and suture, substantially exceed those of native human bladder and ureteral tissue, thereby providing a margin of mechanical safety. The fact that crosslinking alone can elevate tensile strength more than threefold without sacrificing biocompatibility means that a single source material can, in principle, be tuned to different mechanical demands within the urinary tract. Equally important for clinical translation is that the FSB suture’s mechanical superiority is preserved in the wet state, which is the operationally relevant condition for any material deployed in or around the urinary tract, where constant moisture and urine contact would rapidly expose any wet‐state mechanical deficit. The burst pressure and suture retention data from the tubular graft model further reinforce that FSB may tolerate the handling and hemodynamic forces typical of tubular grafting procedures for ureteral and urethral reconstruction.

## 4. The Clinical Landscape of Urological Trauma

Urological trauma and its sequelae represent a significant source of morbidity, with injuries occurring along the entire length of the urinary tract.

### 4.1. Acute Kidney Injury in Urological Practice

Acute kidney injury (AKI) is a significant postoperative complication in urology patients [37]. Several conditions, such as urinary retention, obstructing urinary calculi, and severe urinary tract infections (UTIs), are frequent triggers for AKI [37]. Although the swim bladder is not a direct therapy for the underlying causes of AKI, the high prevalence of this condition underscores the profound vulnerability of the urinary system to injury and highlights the importance of interventions that can support tissue repair and mitigate inflammatory cascades. Renal disease can be induced by any disturbance in the nephron (e.g., in the glomerulus or tubules) [38]. Tubular epithelial cells, particularly proximal tubular cells, have very high mitochondrial/oxidative metabolic demand and are, therefore, vulnerable to ischemic and toxic injury; they are a principal site of acute tubular injury [39–41]. Finally, peritubular capillary integrity and interstitial cell responses—including fibroblast/myofibroblast activation and immune cell crosstalk—strongly influence whether repair proceeds adaptively or progresses to fibrosis [42, 43].

Following AKI, renal tissue initiates a dynamic healing response characterized by inflammation, tubular epithelial cell dedifferentiation and proliferation, extracellular matrix (ECM) production, and compensatory hypertrophy of surviving nephrons [44]. When these processes are well‐coordinated, they allow the kidney to reach a new state of equilibrium and recover function—termed adaptive repair—and this regenerative capacity is thought to underlie the rapid, near‐complete recovery often seen in younger or less severely injured patients [44]. However, the same reparative mechanisms can become excessive, insufficient, prolonged, or misdirected, resulting in maladaptive repair with progressive kidney dysfunction and scarring [44]. Furthermore, persistent G2/M cell‐cycle arrest in tubular epithelial cells can trigger profibrotic signaling and upregulation of TGF‐β, thereby promoting progressive fibrosis and converting AKI to chronic kidney disease (CKD) [44]. Collectively, these findings indicate that the balance between inflammation, epithelial regeneration, and fibrosis determines the outcome of renal repair, emphasizing the importance of interventions that can modulate the inflammatory milieu and support organized tissue remodeling.

### 4.2. Ureteral Injuries in Urological Practice

The ureter is highly susceptible to injury during abdominopelvic surgery. Regardless of the cause, the most feared long‐term complication of ureteral injury is the formation of a fibrotic stricture [45]. Uncontrolled healing may result in excessive scar tissue deposition that narrows and obstructs the ureteral lumen, causing hydronephrosis [46, 47]. The lumen of the ureter is lined by transitional (urothelial) epithelium over a fibro‐elastic lamina propria, followed by a prominent smooth‐muscle coat, and an outer adventitial/serosal layer containing the vascular and neural plexuses [48, 49].

Ureteral damage often leads to disruption of the urothelial lining and inflammation of the lamina propria, which may progress to fibrotic replacement of the tissue and loss of normal smooth‐muscle architecture and peristaltic function [50]. Effective repair, therefore, depends on coordinated re‐epithelialization, restoration of the vascular bed, and regeneration or alignment of the smooth‐muscle layer; failure of these processes typically results in fibrosis and functional impairment. Recent work in ureteral tissue engineering emphasizes scaffolds that provide a matrix supportive of both urothelial and smooth muscle regeneration [51, 52].

### 4.3. Bladder Trauma and Reconstruction

The urinary bladder is also a frequently injured organ, either as a result of surgical procedures or external trauma. The bladder wall is classically described as comprising four principal layers: an innermost layer of urothelium (transitional epithelium), lamina propria, muscularis propria, and outer adventitia/serosa [53, 54]. In response to bladder injury, repair involves rapid restoration of the urothelial barrier, coordinated modulation of inflammation within the lamina propria/stromal compartment, revascularization, and remodeling/regeneration of detrusor smooth muscle [55–57]. The urothelium is now recognized not merely as a passive barrier but as an active sensor and signaling interface, releasing mediators such as adenosine triphosphate (ATP), acetylcholine, and nitric oxide in response to mechanical stretch, chemical stimuli, or injury [55, 58]. This signaling complexity means that damage or disruption of the urothelial barrier can sensitize afferent nerve terminals and provoke inflammatory signaling in the suburothelium [55, 58]. Because the urothelium and lamina propria communicate with the detrusor through paracrine mediators and afferent pathways, sustained barrier compromise and suburothelial inflammation can extend to affect detrusor function [55, 58]. Separately, when suburothelial inflammation persists and ECM progressively accumulates in the detrusor, this is associated with reduced bladder compliance and detrusor overactivity, and can culminate in detrusor fibrosis [56, 59].

### 4.4. Urethral Injury and Stricture Disease

Iatrogenic strictures are most commonly caused by long‐term catheterization or transurethral surgical procedures [60]. Urinary catheters, though essential medical devices, can cause microtrauma that leads to urethral stricture [61–63]. The standard initial treatment, an endoscopic procedure, has a high recurrence rate, trapping patients in a cycle of repeated interventions that potentially worsen the underlying spongiofibrosis [5]. Epithelial thickness varies considerably along the urethra, with the spongy urethra and proximal fossa navicularis exhibiting the thinnest epithelium and lying in proximity to a dense subepithelial vascular network, in contrast to the thicker, keratinized epithelium of the glans and distal fossa navicularis [64]. The male urethra shows regional variation in epithelial lining: the preprostatic and prostatic segments are covered by transitional epithelium (urothelium), the membranous segment is lined by pseudostratified or stratified columnar epithelium, and the spongy urethra—including the meatus—transitions to nonkeratinized stratified squamous epithelium [65, 66]. The penile urethra is mostly surrounded by the corpus spongiosum, a highly vascular spongy tissue that provides mechanical and hemodynamic support to the mucosa [65, 67]. Preservation of the corpus spongiosum during surgical repair is emphasized in reconstructive urology, as this well‐vascularized tissue is associated with improved functional and sexual outcomes [68]. On the other hand, the female urethra is shorter and exhibits a proximal‐to‐distal epithelial gradient, consisting of urothelium near the bladder neck, transitioning to nonkeratinized stratified squamous epithelium distally [66, 69, 70]. In the urethral bed, adequate vascularization of the healing graft is essential, as it supplies the oxygen and nutrients needed for tissue regeneration and supports the formation of a well‐vascularized submucosa; insufficient restoration of this vascularized support contributes to fibrosis and impaired function [71].

When the urethra sustains trauma, the superficial epithelium overlying the corpus spongiosum can be easily breached, exposing the underlying spongy erectile tissue to injury; this disruption may allow urine to extravasate into surrounding tissue, with a resulting risk of infection and inflammation [72]. Experimental models have shown that urinary extravasation into the periurethral mesenchyme induces marked edema and inflammatory cell infiltration [73]. If the epithelial layer fails to regenerate completely or urine exposure persists, fibroblast activation and excessive collagen I deposition occur within the spongiosal tissue, a process termed spongiofibrosis, which culminates in progressive narrowing of the urethral lumen [73, 74]. Urethral stricture disease represents this maladaptive healing response and remains one of the most frequent long‐term sequelae of urethral trauma [72, 74]. Consequently, contemporary management aims to minimize additional tissue injury and inflammation, prevent urine extravasation through adequate urinary drainage or diversion, and optimize conditions for urethral healing while reducing the risk of fibrosis and stricture formation [72]. Emerging regenerative approaches also highlight the potential role of biocompatible scaffolds and ECM‐based hydrogels in supporting tissue regeneration and minimizing fibrotic remodeling [75], suggesting that a similar strategy could potentially be extended to promote urothelial regrowth.

### 4.5. Limitations of Current Biomaterials or Autologous Tissue Grafts

Despite decades of clinical experience, the currently available options for urological reconstruction remain suboptimal. Autologous tissue, particularly buccal mucosa, is regarded as the gold standard for urethral substitution owing to its ease of harvesting, good graft take, and low donor‐site morbidity for short grafts [10]. However, its use is constrained by donor‐site complications, including oral pain, bleeding, swelling, numbness, and impaired speech, mastication, or mouth opening, as well as limited tissue availability [7]. These limitations are further compounded as graft length increases, with acellular grafts showing a critical regenerative threshold as low as 0.5 cm in animal models and uniform clinical failure beyond 4 cm in humans [10].

In the urethra, reliance on buccal or lingual mucosal grafts becomes particularly problematic in long urethral strictures, where adequate donor tissue may not be available without additional morbidity [7, 10]. Nonautologous alternatives, including synthetic scaffolds and acellular matrices such as small intestinal submucosa, have been investigated to overcome the limitations of autologous grafts in urethral reconstruction. However, clinical outcomes remain variable, with reported complications including infection, fistula formation, extravasation, and stricture recurrence, while current evidence remains insufficient to support their routine clinical use [76].

For bladder reconstruction, enterocystoplasty using ileal or colonic segments is a standard surgical option for bladder reconstruction, particularly in patients requiring bladder augmentation [77]. However, this procedure is associated with several metabolic and physiological complications. One of the most common metabolic derangements is hyperchloremic metabolic acidosis, which results from bicarbonate secretion and chloride reabsorption across the intestinal mucosa during prolonged urine contact [77]. This condition can be exacerbated in patients with pre‐existing CKD [78]. Additionally, the use of bowel segments in bladder reconstruction can lead to chronic mucus production, stone formation, and recurrent UTIs [79]. Furthermore, there is a recognized long‐term risk of secondary malignancy associated with enterocystoplasty, as the bowel segments used in the procedure may undergo malignant transformation over time [79, 80].

Reconstruction of the ureter is a complex surgical challenge due to the limited availability of suitable autologous tissue and the associated complications, such as bowel obstruction, metabolic derangement, and recurrent strictures [11, 81, 82]. Recent advancements have introduced alternative techniques, such as buccal mucosal graft (BMG) ureteroplasty and appendiceal onlay/interposition, which are gaining traction for their ability to manage complex ureteral strictures with reduced donor‐site morbidity [11]. BMG, in particular, has shown promise due to its panvascular lamina propria and epithelium, which are well‐suited to the wet environment of the ureter, and has demonstrated a high success rate in ureteral stricture repair without significant complications at the donor site [11, 81]. However, the long‐term durability of BMG and other regenerative approaches remains variable, with reintervention rates increasing over time, reflecting the chronic nature of ureteral stricture disease [83].

On the other hand, FSB‐derived biomaterials may offer a complementary and, in several respects, superior solution to these persistent limitations. Unlike autologous grafts, FSB‐derived matrices are not constrained by donor‐site morbidity or tissue availability, as they can be processed into an “off‐the‐shelf” scaffold independent of harvest from the patient’s own oral mucosa, bladder, or bowel [1, 84]. Structurally, FSB closely approximates the native mammalian ECM, being rich in type I collagen, elastin, and glycosaminoglycans (GAGs) [17], which together confer mechanical resilience, hydration, and bioactivity that are favorable for cellular adhesion and constructive tissue remodeling [75]. These intrinsic properties may, by extension, be advantageous in moist, urine‐exposed environments such as the urethra or ureter, although this application‐specific potential remains to be empirically validated. Furthermore, as a xenogeneic, nonmammalian biomaterial, FSB carries a substantially lower risk of zoonotic disease transmission than bovine‐ or porcine‐derived ECM products, while also circumventing the religious and cultural restrictions associated with mammalian tissue sourcing [16, 85]. From a translational and sustainability perspective, FSB is derived from an abundant fish‐processing by‐product that is otherwise discarded as waste, enabling the value‐added utilization of marine biomass while reducing the environmental burden and providing a readily available source of collagen for biomaterial development [15, 27, 86, 87].

Taken together, these structural, immunological, and logistical advantages position FSB‐derived biomaterials as a promising “off‐the‐shelf” platform capable of addressing the core limitations of current urological reconstructive options—donor‐site morbidity, limited graft availability, and intestinal‐tissue‐related complications—while warranting further investigation to establish their clinical efficacy and long‐term safety in human urological applications.

## 5. Bioactive Constituents of Fish Swim Bladder and Their Roles in the Wound‐Healing Process

The regenerative potential of the FSB is primarily attributed to its complex and biologically active biochemical composition. It is not an inert substance but a dynamic matrix containing multiple bioactive components that can actively and favorably influence the key phases of wound healing.

### 5.1. Type I Collagen as the Principal Bioactive Component of the Swim Bladder

The swim bladder contains valuable components, most notably type I collagen, which is the predominant form of collagen in the body [17]. Type I collagen consists of two *α*1 chains and one *α*2 chain, which are left‐handed polypeptides twisted together into a right‐handed triple helix [17]. Each chain contains roughly 1014 amino acids, with glycine as the most dominant (approximately 30%), followed by proline, hydroxyproline, threonine, methionine, isoleucine, and phenylalanine [17]. Although standard sources of collagen generally come from cattle, horses, poultry, or pigs [88–90], there are several drawbacks to using animal‐derived collagen products—including the risk of allergic reactions, the spread of zoonotic diseases (such as bovine spongiform encephalopathy and transmissible spongiform encephalopathy), and microbial contamination [91, 92]. Additionally, in certain communities, there are religious restrictions on the use of tissues derived from cattle and pigs [85, 93]. As an alternative, marine sources are being studied because they are considered safer than those from terrestrial animals [94]. Marine‐derived collagen offers several advantages—including easier extraction, greater chemical and physical stability, high biodegradability, good biocompatibility, and low immunogenicity [94]. Moreover, marine collagen is an abundant raw material, often regarded as “waste” in the fish processing industry, and can be recycled into collagen‐based products [17, 95].

However, it should be noted that some types of marine collagen may denature at temperatures below the human physiological temperature [96]. This thermal instability can present practical challenges in its use, particularly for medical applications in humans [96]. This can be mitigated by sourcing from warm‐water species or by utilizing the material as a decellularized matrix, where the native cross‐linking preserves its structural integrity at physiological temperatures [97].

Studies have demonstrated that type I collagen extracted from marine swim bladders can enhance wound healing. An experimental study conducted by Li et al. on ICR mice showed that type I collagen, which is present in the swim bladder of the sea eel (*Muraenesox cinereus*) as a pepsin‐solubilized collagen (PSC) sponge, can significantly accelerate wound healing and reduce inflammation severity in the initial phase of wound healing [98]. Additionally, an experimental study by Chen et al. in ICR mice found that the giant croaker (*Nibea japonica*) swim bladder, as a PSC sponge, significantly promoted wound healing [99]. Another organ source besides swim bladders that can potentially be applied in urological reconstruction is described in an experimental study using a rabbit urethral defect model, in which Huang et al. proved that decellularized tilapia skin significantly increased tissue collagen and smooth muscle content, resulted in a significantly milder inflammatory response in urethral defect healing, and promoted better angiogenesis and wound healing, without significant immune rejection during urethral reconstruction [100]. Experimental studies by Lv et al. on Sprague–Dawley rats [101] and Wang et al. on BALB/c mice and Bama pigs [102] using tilapia skin acellular dermal matrix (TADM) with type I collagen as the main component demonstrated that TADM significantly promoted wound healing through several mechanisms (i.e., upregulating growth factor expression and enhancing angiogenesis, collagen deposition, and tissue regeneration) [101, 102]. On the other hand, experimental studies by Ge et al. on Sprague–Dawley rats [103] and Im et al. on BALB/c mice [104] showed that tilapia skin‐based hydrogel significantly accelerated the healing process of second‐degree burn wounds [103] and even full‐thickness wounds [104]. Thus, TADM and tilapia skin‐based hydrogel serve as promising novel wound dressings. In addition, an experimental study by Wang et al. demonstrated a promising urinary diversion alternative, using decellularized FSB seeded with adipose‐derived stem cells and bladder smooth muscle cells isolated from rabbits to form tissue‐engineered tubular structures (TETSs), which showed progressive maturation of multilayer luminal urothelium, neovascularization, and smooth muscle regeneration, along with the absence of urinary leakage and no stricture or obstruction in the TETSs or ureter [84].

#### 5.1.1. Mechanism 1 (Hemostasis Initiation and Modulation of the Inflammatory Response)

The wound‐healing cascade begins with hemostasis [105]. Upon injury, the exposure of collagen fibers in the subendothelial matrix provides a potent stimulus for platelet activation and aggregation, leading to the formation of a primary hemostatic plug and a fibrin clot [105]. Immediately following this, the inflammatory phase begins [105]. This initial inflammatory response is crucial for proper healing [73, 106]. However, prolongation of this phase can cause fibrosis [73, 105, 106]. Collagen derived from FSB appears to modulate the local inflammatory response by promoting macrophage polarization toward an anti‐inflammatory M2 phenotype while limiting excessive inflammatory cell infiltration [75]. This immunomodulatory effect may facilitate the timely resolution of inflammation, thereby creating a proregenerative microenvironment that supports tissue repair and minimizes fibrotic remodeling [75]. Furthermore, recent evidence suggests that fish‐derived collagen can beneficially modulate the immune response by polarizing macrophages toward the M2, or “pro‐regenerative,” phenotype [107]. M2 macrophages are critical for resolving inflammation, promoting angiogenesis, and supporting tissue remodeling, in contrast to the proinflammatory M1 phenotype associated with chronic wounds [107]. This immunomodulatory property is a key mechanism by which swim bladder collagen may help prevent the development of fibrotic scar tissue. The complete mechanism is shown in Figure 2.

**FIGURE 2:**
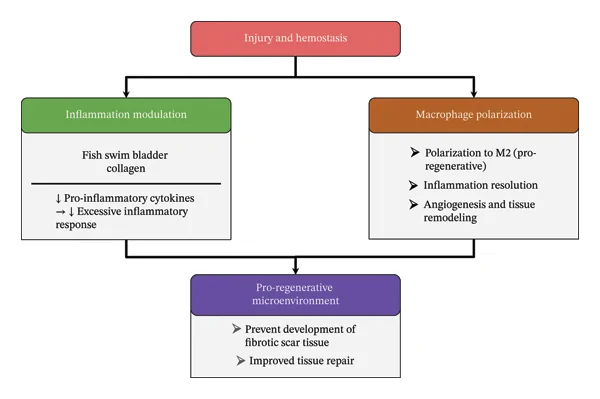
Hemostasis initiation and modulation of the inflammatory response.

#### 5.1.2. Mechanism 2 (Angiogenesis and Re‐Epithelization)

Beyond its structural role, the C‐propeptide fragment of type I collagen has also been shown to act as a chemoattractant for endothelial cells, potentially triggering angiogenesis during wound healing [108]. This process is crucial for delivering oxygen, nutrients, and growth factors to regenerating tissue [108]. Biomaterials, such as FSB‐derived type I collagen, appear to support this vascular and nutrient‐delivery phase [15]. At the same time, collagen degradation fragments promote the synthesis of growth factors that, together with fibroblast proliferation, contribute to re‐epithelialization [109]. The complete mechanism is shown in Figure 3.

**FIGURE 3:**
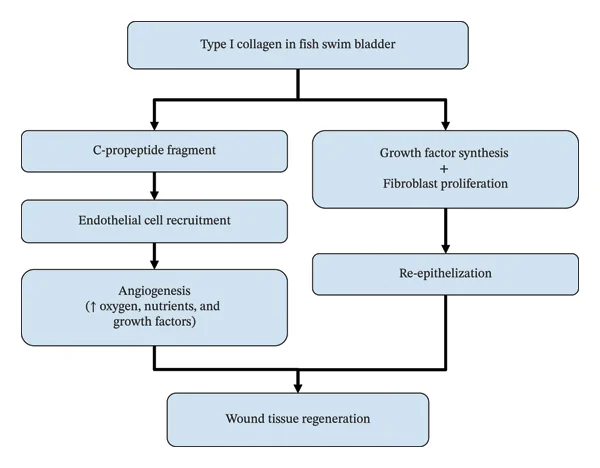
Angiogenesis and re‐epithelization.

#### 5.1.3. Mechanism 3 (Regulation of Matrix Metalloproteinase Activity)

A key feature of chronic, nonhealing wounds is an imbalanced microenvironment characterized by excessive levels of degradative enzymes, particularly matrix metalloproteinases (MMPs) [109]. These enzymes degrade deposited collagen and other ECM components, thereby disrupting matrix remodeling and preventing progression toward normal tissue repair [109]. When a collagen‐based biomaterial, such as the FSB matrix, is applied to the wound, it may act as a sacrificial substrate (or “decoy”) for excess MMPs, thereby protecting newly deposited ECM from excessive proteolytic degradation [16, 110]. Collagen‐based scaffolds have been reported to modulate the inflammatory response while promoting fibroblast proliferation, angiogenesis, and re‐epithelialization, thereby supporting progression through the wound‐healing process [16, 111, 112]. The complete mechanism is shown in Figure 4.

**FIGURE 4:**
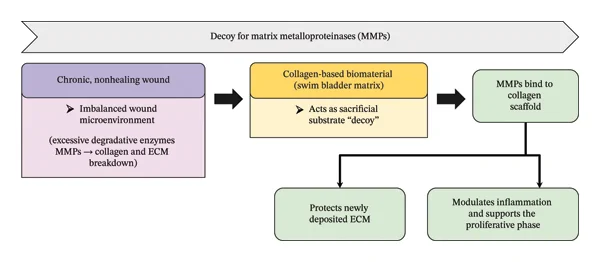
Regulation of matrix metalloproteinase activity.

### 5.2. Antioxidant Activity of Fish Swim Bladder–Derived Components in Oxidative Stress Modulation

Injured and inflamed tissues exhibit intense metabolic activity, leading to the generation of large quantities of reactive oxygen species (ROS) [113]. In physiological concentrations, ROS are important signaling molecules involved in antimicrobial defense, platelet activation, vasoconstriction, angiogenesis, and ECM remodeling [114]. However, when present in excess, ROS induce oxidative stress (OS), which exacerbates inflammation and disrupts healing, leading to chronic wounds, ongoing inflammation, and defective tissue repair [114]. Antioxidants act through various mechanisms—including direct scavenging of ROS, inhibition of ROS precursors, and enhancement of endogenous defense systems [114]. Antioxidants have shown therapeutic potential in reducing OS by neutralizing excess ROS, thereby preventing prolonged inflammation during the inflammatory phase of wound healing, avoiding impaired angiogenesis, and accelerating epithelialization and tissue repair [114].

Experimental studies conducted by Zhao et al. and Chen et al. demonstrated that type I collagen derived from the swim bladder of miiuy croaker [115] and giant croaker [99], encompassing both acid‐soluble collagen (ASC) and pepsin‐soluble collagen (PSC), exhibited antioxidant capacity to scavenge free radicals such as 2,2‐diphenyl‐1‐picrylhydrazyl (DPPH), hydroxyl radical, superoxide anion, and 2,2′‐azino‐bis‐3‐ethylbenzothiazoline‐6‐sulfonate (ABTS) in a dose‐dependent manner [99, 115]. The findings were consistent with an experimental study by Dong et al., in which type I collagen extracted from the swim bladders of grass carp, bighead carp, grouper, and monkfish scavenged DPPH and ABTS radicals [116]. Jiang et al. also demonstrated that type I collagen from the silver carp swim bladder exhibited DPPH radical‐scavenging activity [97]. These activities helped to reduce OS, which is known to worsen inflammation and hinder mucosal healing.

Although the literature on the direct role of antioxidants derived from FSB in the urinary tract mucosa remains limited, there is strong evidence that OS and ROS contribute to the functional and structural pathology throughout the lower urinary tract, providing a biological basis for antioxidant strategies targeting the urinary tract [117–120]. Studies have shown that excessive ROS, often arising from ischemia and ischemia‐reperfusion injury, can trigger functional and structural changes in the urethra and urinary bladder—including disruption of tissue integrity, chronic inflammation, and vascular impairment—contributing to lower urinary tract dysfunction and symptom development [117–119]. In an experimental study by Öğüş et al. on Wistar‐Albino rats with urethral injury, total oxidant status (TOS) levels increased nearly fourfold in the penile and bladder on the first day postinjury, reflecting a surge of ROS during the early inflammatory phase [120]. TOS levels in the penile and bladder were reduced by almost 50% 7 days postinjury, but continued to decline toward control values in the third week for the bladder TOS level, while remaining high for the penile tissue TOS level [120]. For total antioxidant capacity (TAC) values, the bladder TAC value increased slightly on the first day, then progressively declined to half the control TAC value by the third week [120]. However, the penile TAC value increased twofold on the first day and stayed high until the third week [120].

### 5.3. Polysaccharides as Microenvironment Modulators

In addition to collagen, polysaccharides (up to 10%) as FSB components, primarily GAGs, have been known to play a vital role in modulating the wound microenvironment [17, 121].

#### 5.3.1. CS

Comprising approximately 95% of the total polysaccharides in the swim bladder, CS plays a significant role during the process of wound healing [17, 121]. An in vitro study by Zou et al. on cultured rabbit palatal fibroblast tissue showed that administration of chondroitin‐6‐sulfate in a dose‐dependent manner enhanced cell adhesion and fibroblast proliferation—both of which are crucial in the proliferative phase of wound healing [122]. Conversely, when CS was degraded by the enzyme chondroitinase, cell adhesion and proliferation decreased, functionally slowing wound closure in culture [122].

#### 5.3.2. HA

Although it constitutes a smaller portion (around 5%) of the polysaccharides, HA has a disproportionately significant impact on wound healing [17, 123]. As a highly hydrophilic molecule, HA is critical for maintaining a hydrated wound environment, which is essential for facilitating cell migration (i.e., keratinocytes and fibroblasts) and accelerating re‐epithelialization [123]. Beyond this physical role, HA is a potent signaling molecule that binds cellular receptors such as CD44, activating intracellular pathways involved in cell adhesion, migration, and proliferation [124]. HA is CD44’s primary and most specific ligand, and both are upregulated after skin injury, making HA‐CD44 signaling a regulator across all four phases of wound healing (hemostasis, inflammation, proliferation, and remodeling) [124]. Crucially, the functional outcome of this interaction hinges on HA’s molecular weight [124]. High molecular weight HA (HMW‐HA, > 1000 kDa) cross‐links CD44 into clusters, producing anti‐inflammatory and antifibrotic effects that stabilize the ECM, drive macrophages toward the M2 phenotype, and favor regenerative healing [124]. Whereas low molecular weight HA (LMW‐HA, < 500 kDa) cleaves cross‐linked CD44 and signals through monomeric CD44, driving proinflammatory and profibrotic responses that favor scarring [124]. Jalali et al. demonstrated that in a controlled rat wound‐healing experiment using acellular matrix derived from the white‐fish (*Rutilus frisii kutum*) swim bladder impregnated with HA, the combined treatment resulted in significantly accelerated wound closure compared with either the matrix alone, HA alone, or the untreated control [125]. Histologically at day seven, the acellular FSB matrix impregnated with HA group displayed reduced inflammatory cell density, increased fibroblast cell synthesis, enhanced collagen synthesis, and a higher rate of angiogenesis, indicating that native swim bladder matrix components and HA act synergistically to create an optimized regenerative microenvironment [125].

## 6. Antimicrobial Effects

In urology, one of the most frequent clinical problems associated with infection is UTI, which can ascend from the urethra and cause cystitis or even pyelonephritis [126]. Recurrent infection, biofilm formation, multidrug resistance, and device‐associated colonization are key challenges in the urological milieu [127]. Given this infection burden, the idea of swim bladder–derived biomaterial has fascinating antimicrobial potential.

The FSB, commonly processed into gelatin or collagen, represents a valuable biomedical material due to its abundance of type I collagen, high biocompatibility, and excellent film‐forming properties [17]. These characteristics make it a promising substrate for swim bladder‐derived biomaterial use. While collagen itself is not inherently bactericidal, enzymatic hydrolysis of collagen can generate collagen‐derived peptides with bioactive properties, including antioxidant activity [27, 128, 129]. Furthermore, broader groups of antimicrobial peptides (AMPs), such as cathelicidins, β‐defensins, hepcidins, and piscidins, are well characterized in fish. It has been shown that in some species of fish that have been tested, AMPs (i.e., β‐defensins and piscidins) were present in the structural organs of fish, one of which is the swim bladder [130–132]. The widespread presence of β‐defensins and piscidins across fish tissues suggests they contribute to antimicrobial activity.

AMPs are essential components of the urinary tract’s innate immune defense [133–135]. Produced by the urothelium, they serve as frontline antimicrobial and immunomodulatory mediators [133–135]. In humans, defensins are a class of small (2–5 kDa), cysteine‐rich cationic peptides that contain three intramolecular disulfide bonds, providing structural stability and reducing protease‐mediated degradation [136, 137]. Defensins exhibit direct antibacterial/antiviral effects, largely via membrane‐disruption mechanisms [136, 137] and have been characterized in teleost fish, where they display antimicrobial and immunomodulatory functions [138]. Human β‐defensin‐1 (hBD‐1) is constitutively expressed in the kidney, ureter, and bladder epithelium, indicating a baseline antimicrobial presence across the urinary tract [134, 139]. hBD‐1 can be detected in urine and is thought to act by forming a chemical barrier that inhibits bacterial attachment to urothelial surfaces [134, 139, 140]. In contrast, human β‐defensin‐2 (hBD‐2) demonstrated inducible expression in the urinary tract, with significantly elevated urinary levels reported in inflammatory bladder disease (e.g., ulcerative interstitial cystitis) [141] and increased expression in chronic upper UTI [142], supporting its role as a responsive AMP upregulated during mucosal stress. A bladder epithelial cell study further showed that infection‐related signaling pathways modulated hBD‐1 (with hBD‐2 undetected) expression and secretion, as nucleotide oligomerization domain‐like receptor pyrin domain‐containing 3 (NLRP3) activity is required to maintain AMP output and effective epithelial antibacterial function against uropathogenic *Escherichia coli* [133]. Loss of NLRP3 signaling reduces hBD‐1 secretion and impairs the antimicrobial capacity of bladder epithelial secretions, underscoring the pathway’s importance in sustaining AMP‐mediated defense [133]. Mechanistically, AMPs are positively charged due to the presence of lysine and/or arginine residues and are amphipathic, enabling them to accumulate at high concentrations in both aqueous environments and biological membranes [143]. AMPs exert their antimicrobial effects by rapidly targeting microbial membranes, leading to microbial death within seconds to minutes [143]. Although AMPs exhibit diverse structural characteristics, their antimicrobial activity is preserved as long as they maintain their cationic charge and amphipathic properties [143]. While β‐defensins are conserved across vertebrates, interspecies variation is evident; a study in teleost fish illustrated structural diversity within the defensin family, reinforcing their evolutionary breadth and functional adaptability [138]. In the context of UTIs, urinary β‐defensin levels have been shown to correlate with infection risk and symptom severity, suggesting that alterations in defensin expression may influence susceptibility [144].

Piscidins, a family of fish‐derived AMPs, employ a multifaceted mechanism of antimicrobial activity [130, 145, 146] that could be leveraged in urological biomaterials. Piscidins are small, cationic, amphipathic α‐helical peptides that kill bacteria by directly attacking the cell membrane [130, 145, 146]. The peptide’s cationic, hydrophilic face lets it interact with acidic phospholipids, assembling into toroidal pores that breach the pathogen membrane [130]. The biophysical comparison of piscidin‐1 (p1) and piscidin‐3 (p3) showed that the degree of this damage depends on histidine content and its protonation state, where p1 carries an extra histidine that stays largely neutral even at acidic pH, allowing it to insert more deeply and at a steeper tilt into the bilayer—its C‐terminus reaching into the opposing leaflet—producing greater membrane thinning, structural defects, and altered membrane conductance than p3, which loses much of its permeabilizing activity once the environment turns acidic [145]. Moreover, a piscidin–glycolipid combination study reinforced the same membrane‐targeted mode of action, showing that p1 and p3 transform into their active α‐helical conformation upon binding a yeast‐derived glycolipid (using sophorolipid‐hexyl ester/SL‐HE as a modified yeast‐derived glycolipid biosurfactant), after which the peptide–glycolipid accumulates on the target membrane and disrupts it far more severely than either the peptide or the glycolipid does alone, with the glycolipid additionally functioning to deliver and recruit the peptide to the bacterial membrane [146]. The piscidin family of AMPs is primarily found in teleost fish, with multiple isoforms and transcripts that have been studied and have played a broad‐spectrum role in innate immunity across various fish tissues—including gills, skin, kidney, swim bladder, and gut [132, 147–149]. Piscidins (p1 and p3) carry an N‐terminal ATCUN (Amino Terminal Cu^2+^/Ni^2+^‐binding) motif that binds copper and nickel ions with high affinity. Molecular dynamics simulations comparing metal‐free and nickel‐bound P1 and P3 showed that nickel binding made the ATCUN motif more conformationally rigid and reduced the N‐terminal charge, allowing piscidins to embed deeper in the bilayer to enhance antimicrobial activity [150]. Moreover, piscidins have been shown to inhibit biofilm formation by pathogenic Gram‐negative organisms, such as *Escherichia coli*, by disrupting membrane integrity and interfering with biofilm formation [151]. Although direct studies of piscidins in UTIs are limited, their broad‐spectrum activity—including relevant uropathogens and potent membrane‐disruptive and antibiofilm capabilities—suggests translational potential in the urinary tract environment. The antimicrobial capacity of swim bladder‐based biomaterials, therefore, appears limited when used in their native form. An in vitro study by Howaili et al. demonstrated significant antibacterial activity using disc diffusion and growth inhibition methods, based on a novel antimicrobial wound dressing derived from the swim bladder of *Rutilus frisii*, doped with silver nanoparticles (AgNPs) [152]. Similarly, an in vitro study by Ferreira et al. combined chitosan with fish collagen extracted from the swim bladder of *Hexanematichthys parkeri*, producing polymer blends that inhibited microbial activity [153].

Taken together, the evidence suggests that the intrinsic antimicrobial effects of swim bladder‐derived biomaterials are minimal, and functionalization with antimicrobial compounds is necessary for clinically meaningful protection. Within this landscape, swim bladder‐derived biomaterials are most realistically positioned as a biocompatible base matrix rather than a standalone antimicrobial solution.

## 7. Safety and Biocompatibility of Swim Bladder–Derived Biomaterials on Urinary Tract

Decellularized swim bladder scaffolds and derived fish‐collagen preparations have demonstrated strong mechanical properties, retention of a highly organized three‐dimensional fibrillar network, and elicited a constructive remodeling response rather than a chronic foreign‐body reaction in several in vivo models [17, 154].

### 7.1. Allergy, Immunogenicity, Sterilization, and Processing Controls

A central safety question for any fish‐derived biomaterial is allergenicity: although the major known fish allergen is parvalbumin, increasing evidence indicates that fish collagen (and other ECM/matrix proteins) can also evoke IgE‐mediated hypersensitivity in predisposed individuals [155–157]. Severe allergic reactions to fish collagen and its derivative products, including anaphylaxis, have been reported [155–157]. In addition, occupational exposure to aerosolized fish proteins during seafood processing has been associated with respiratory and cutaneous allergic symptoms, highlighting the need for careful allergenic risk assessment of fish‐derived materials [155–157]. The immunogenic potential of fish‐derived collagen is influenced by the species of origin and the quality of the extraction and processing procedures [15, 17]. Effective removal of noncollagenous proteins and other residual impurities during collagen extraction can improve the purity and biocompatibility of the final material [15, 17]. Indeed, reviews of fish collagen biomaterials emphasize that the physicochemical properties and biological performance of collagen are influenced by both the fish species and the extraction and purification procedures employed, and experimental studies on FSB‐derived scaffolds have demonstrated favorable in vivo biocompatibility, with optimized extraction and purification procedures considered important for obtaining high‐quality biomaterials suitable for biomedical applications [15, 17].

A recent study on FSB‐derived biomaterials reported no organ toxicity and minimal aberrant immune responses in vivo following decellularization [158], and broader studies of marine collagen‐ and ECM‐based biomaterials consistently report favorable biocompatibility and low immunogenicity, while emphasizing that appropriate extraction, purification, and cross‐linking procedures are important for optimizing biomaterial performance [17]. Overall, patient screening and clear product labeling will be essential for safe clinical translation.

### 7.2. Infectious Disease and Contamination Risk

As with any biologic tissue‐derived scaffold, appropriate processing is required to minimize microbial contamination risk [159, 160]. Fish‐derived ECM scaffolds inherently avoid the risk of mammalian prion transmission associated with mammalian tissues; however, rigorous processing and sterilization remain essential to minimize microbial contamination and other process‐related contaminants [159]. Effective disinfection of FSB ECM using 75% ethanol has been demonstrated, with complete removal of culturable bacteria and preservation of collagen, elastin, and GAG architecture and mechanical function following rinsing [160]. Multiple sterilization or disinfection methods, such as ethanol, peracetic acid, ethylene oxide, and irradiation, have been applied to decellularized ECM scaffolds, and method selection must balance sterility assurance with retention of biological activity and ultrastructure [159]. Therefore, sterilization and validation steps must be integrated into manufacturing pipelines for any ECM‐based product intended for clinical use [159]. Confirmation of effective sterilization, together with evaluation of scaffold integrity and biocompatibility, is essential to ensure the safety of ECM scaffolds before clinical application [159].

## 8. Impacts of Source Species on Fish Swim Bladder–Derived Biomaterials

Although this review presents several lines of evidence across numerous fish species, an important variable that warrants explicit discussion is the extent to which the source species itself dictates the physicochemical and mechanical performance of FSB‐derived biomaterials and, by extension, their suitability for urological applications. Several lines of evidence support measurable species‐dependent variation in FSB composition. Dong et al. directly compared PSC extracted from the swim bladders of four fish species—grass carp, bighead carp, grouper, and monkfish—and reported substantial differences in extraction yield (38.98%, 27.97%, 18.16%, and 10.35%, respectively), attributed to differences in swim bladder matrix compactness, degree of native cross‐linking, and species‐specific biology [116]. The same study found that amino acid contents, a key determinant of collagen triple‐helix and collagen’s thermal stability, were highest in grass carp and lowest in monkfish, a pattern linked to the warm‐versus cold‐water habitats of the source species [116]. On the other hand, based on the dry weight of swim bladders, the extraction yield shown by Zhang et al. for Bester sturgeon was 37.7% [161]; Ghani et al. reported yields for carp and Bester sturgeon of 17.16% and 24.88%, respectively [162]; and Jiang et al. reported yields for silver carp and grass carp of 5.92% and 3.92%, respectively [97].

Thermal stability appears to be the species‐dependent property of direct relevance to urological applications. As noted earlier in this review, FSB collagen has a thermal denaturation temperature of approximately 35°C [17]. Species‐level data verify this variability, where in the four‐species comparison above, bighead carp swim bladder collagen, a warm‐water species, exhibited the highest thermal denaturation temperature (38.60°C)—exceeding human core body temperature (or physiological temperature)—while monkfish, a cold‐water species, exhibited the lowest (29.85°C) [116]. Silver carp swim bladder collagen had also been highlighted for its high thermal denaturation temperature (40.08°C) and thermostability compared to other fish‐derived collagens [97]. Swim bladder from several other species that had lower thermal denaturation temperatures than human core body temperature were carp (34.78°C) [162], grouper (33.84°C) [116], giant croaker (33.8°C) [99], totoaba (32.5°C) [27] and Bester sturgeon (31.75°C) [162]. A collagen source whose native denaturation temperature sits below or only marginally above body temperature risks progressive loss of triple‐helical integrity—and consequently mechanical strength—once implanted [96, 97].

Moreover, species‐dependent differences in gel‐forming and viscoelastic behavior that are directly relevant to handling and structural performance need to be highlighted. An experimental study by Mredha et al. showed that Bester sturgeon swim bladder collagen exhibited rapid fibrillogenesis, enabling it to form macroscopically oriented, fiber‐bundled hydrogels via diffusion‐induced gelation [19]. Another experimental study by Ghani et al. found that carp swim bladder formed more transparent, mechanically stronger, and more viscous gels than Bester sturgeon swim bladder, even though Bester sturgeon collagen formed fibrils faster within 3 h of gelation, versus 24 h for bundle formation in carp collagen [162]. As the gelation process continued, collagen fibrils gradually became thicker and shorter [162]. Bester sturgeon collagen generated fibrils that were initially more elongated, thicker, and better aligned than those produced from carp collagen, although their diameter continued to increase with extended gelation [162]. By contrast, carp collagen showed fewer fibrils and a rougher surface after 3 h of gelation, indicating that fibril formation had started but had not yet reached completion [162]. Continued gelation promoted rapid fibril development, and fibrillar bundles became evident for carp collagen after 24 h of gelation [162]. The formation of thicker fibrils coincided with a decline in fibril number, thereby reducing fibrillar connectivity and yielding a gel with reduced mechanical rigidity [162]. Additionally, the gelation process can substantially increase the thermal denaturation temperature, which is proportional to the extent of fibril formation [19, 162].

Complementarily, recent studies have demonstrated the fabrication of FSB‐derived collagen into surgical sutures. An experimental study by Luan et al. developed an absorbable suture from grass carp swim bladder that resists high hydrostatic pressure and acidic conditions [36]. In a simulated gastric environment (pH 1.0, up to 7 days), the DCSS suture tensile strength stayed statistically unchanged, while catgut’s tensile strength dropped steadily [36]. A study conducted by Sun et al. investigated an absorbable suture derived from the swim bladder of freshwater goldfish (*Carassius auratus*). The researchers functionalized the sutures with either rapamycin (RAPA) or necrostatin‐1 (NEC‐1) using coatings of HA and polyvinyl alcohol. The results showed that all suture variants—control, RAPA, and NEC‐1—maintained their structural integrity in both strongly acidic (pH 1.0) and alkaline (pH 8.0) conditions for at least 7 days [163]. However, the tensile strength of the NEC‐1 and RAPA sutures remained consistently higher than that of the control sutures, potentially due to the presence of the HA coating [163]. Although neither study was conducted in a urological model, this demonstrated tolerance is notable given that human urine pH typically fluctuates between approximately 4.5 and 8, and can shift in urological disorders, such as infection or metabolic disturbance [164, 165], suggesting that FSB collagen may be intrinsically suited to withstand the variable luminal environment of the urinary tract.

Collectively, these findings indicate that fish species are a critical determinant of the physicochemical, thermal, and gelation characteristics of FSB‐derived biomaterials, thereby directly influencing their mechanical performance and potential suitability for urological tissue engineering. Variations in fish habitat ecology, collagen yield, imino acid content, thermal denaturation temperature, fibrillogenesis, and gelation behavior indicate that the biological origin of the swim bladder substantially influences scaffold performance. Consequently, species selection should be regarded as an essential criterion during biomaterial development rather than merely a source of raw collagen. The available evidence permits a cautious, hypothesis‐generating inference rather than a definitive species recommendation. Instead, the optimal species will likely depend on the intended clinical application and the balance between thermal stability, scaffold mechanics, processability, and biological performance. It must be emphasized, however, that no published study to date has directly benchmarked FSB mechanical or chemical properties across multiple species against the specific biomechanical or biochemical demands in the urinary tract—such as burst pressure, suture retention strength, compliance matched to native detrusor or corpus spongiosum tissue, degradation resistance under chronic exposure to urine and urease‐producing uropathogens, or pH tolerance.

## 9. Limitations

Although various preclinical studies have demonstrated that FSB‐derived biomaterials show favorable biocompatibility and low immunogenicity, important translational issues—including long‐term biodegradation, performance under chronic urine exposure, resistance to infection and biofilm formation, biomechanical and biochemical variability, and allergenicity—remain insufficiently investigated in the urological field. Most available evidence remains limited to in vitro and animal studies. Clinical evidence regarding the safety and efficacy of FSB‐derived biomaterials in humans remains unavailable. Current research is largely restricted to basic biological aspects—including biocompatibility, wound‐healing potential along with antioxidant activity, antimicrobial activity, and immune responses—suggesting that clinical validation remains necessary. Furthermore, heterogeneity exists among fish species regarding collagen content, thermal stability, gelation behavior, and mechanical properties, while differences in extraction, decellularization, crosslinking, and sterilization protocols may hinder standardization. Finally, the intrinsic antimicrobial activity of unmodified FSB‐derived biomaterials appears minimal, meaning that clinically meaningful protection would depend on further functionalization.

## 10. Conclusions and Future Directions

FSB‐derived biomaterials demonstrate considerable promise as a marine‐based ECM platform for urological tissue engineering, owing to their favorable structural composition, biocompatibility, and regenerative potential. Current evidence indicates beneficial roles in wound healing and tissue remodeling, although intrinsic antimicrobial activity remains limited and often requires functional enhancement. Safety concerns, including allergenicity and processing variability, must be carefully addressed to ensure clinical applicability. However, successful clinical translation will require standardized manufacturing and quality‐control protocols, optimization of species selection, and comprehensive preclinical validation in urological models. Future research should prioritize standardized manufacturing, long‐term safety evaluation, biomechanical and biochemical performance, and compatibility within the urinary environment to facilitate successful clinical translation and to deliver safe, effective, and standardized products suitable for clinical use.

## Funding

This study received no external funding.

## Conflicts of Interest

The authors declare no conflicts of interest.

## Data Availability

Data sharing is not applicable to this article, as no datasets were generated or analyzed during the current study.
